# Screening, brief intervention, and referral to treatment (SBIRT) implementation models and work flow processes: commonalities and variations

**DOI:** 10.1186/1940-0640-8-S1-A79

**Published:** 2013-09-04

**Authors:** Janice Vendetti, Bonnie McRee, Amy Hernandez, Georgia Karuntzos

**Affiliations:** 1 Health Services Research Unit, Department of Community Medicine and Health Care, University of Connecticut Health Center, Farmington, CT, USA; 2 Behavioral Health and Criminal Justice Research Division, RTI International, Research Triangle Park, NC, USA

## Introduction

The Substance Abuse and Mental Health Services Administration (SAMHSA) sponsored a cross-site evaluation of the grantees of their Screening, brief intervention, and referral to treatment (SBIRT) program for alcohol and other drugs funded in 2008. As part of this evaluation, the cross-site evaluation team was tasked with understanding the implementation and service delivery protocols and work flow processes related to SBIRT service delivery. SBIRT programs have been implemented in a variety of different settings and performance sites, and each has adjusted the flow of SBIRT services to meet the needs of the particular setting**.** This presentation will focus on the variations in SBIRT program activities and work flow by three performance site types: (1) Emergency Departments; (2) Inpatient Hospitals; and (3) Outpatient Clinics. Descriptions of the service providers involved in the various SBIRT service components are presented**.**

## Methods

Findings were based primarily on observations of program delivery and reviews of grantee-provided data sources (e.g., grant applications, annual reports, site visit notebook materials), were supplemented with the qualitative analyses of semi-structured interviews with key SBIRT staff. Data extraction techniques were used to review the grantee-provided data sources. Qualitative analyses were conducted using ATLAS.ti software to examine relevant code combinations regarding SBIRT service delivery components and service provider characteristics.

## Results

The synthesis of the grantee based document review data and qualitative analyses of semi-structured interview data will be presented. Work flow charts will be developed by performance site type depicting the most common work flow elements with variations in separate boxes. Commonalities and variations in SBIRT work flow processes will be discussed in this presentation**.**

## Conclusions

Based on the “typical” SBIRT work flow processes, recommendations for initial program development will be discussed for those seeking to establish SBIRT services within various settings and performance sites**.**

